# Proteome analysis of *Aspergillus niger*: Lactate added in starch-containing medium can increase production of the mycotoxin fumonisin B_2 _by modifying acetyl-CoA metabolism

**DOI:** 10.1186/1471-2180-9-255

**Published:** 2009-12-10

**Authors:** Louise M Sørensen, Rene Lametsch, Mikael R Andersen, Per V Nielsen, Jens C Frisvad

**Affiliations:** 1 Department of Systems Biology, Søltofts Plads, Technical University of Denmark, DK-2800 Kgs Lyngby, Denmark; 2 Department of Food Science, Faculty of Life Sciences, University of Copenhagen, Rolighedsvej 30, DK-1958 Frederiksberg C, Denmark; 3 IPU, Produktionstorvet, building 425, DK-2800 Kgs Lyngby, Denmark

## Abstract

**Background:**

*Aspergillus niger *is a filamentous fungus found in the environment, on foods and feeds and is used as host for production of organic acids, enzymes and proteins. The mycotoxin fumonisin B_2 _was recently found to be produced by *A. niger *and hence very little is known about production and regulation of this metabolite. Proteome analysis was used with the purpose to reveal how fumonisin B_2 _production by *A. niger *is influenced by starch and lactate in the medium.

**Results:**

Fumonisin B_2 _production by *A. niger *was significantly increased when lactate and starch were combined in the medium. Production of a few other *A. niger *secondary metabolites was affected similarly by lactate and starch (fumonisin B_4_, orlandin, desmethylkotanin and pyranonigrin A), while production of others was not (ochratoxin A, ochratoxin alpha, malformin A, malformin C, kotanin, aurasperone B and tensidol B). The proteome of *A. niger *was clearly different during growth on media containing 3% starch, 3% starch + 3% lactate or 3% lactate. The identity of 59 spots was obtained, mainly those showing higher or lower expression levels on medium with starch and lactate. Many of them were enzymes in primary metabolism and other processes that affect the intracellular level of acetyl-CoA or NADPH. This included enzymes in the pentose phosphate pathway, pyruvate metabolism, the tricarboxylic acid cycle, ammonium assimilation, fatty acid biosynthesis and oxidative stress protection.

**Conclusions:**

Lactate added in a medium containing nitrate and starch can increase fumonisin B_2 _production by *A. niger *as well as production of some other secondary metabolites. Changes in the balance of intracellular metabolites towards a higher level of carbon passing through acetyl-CoA and a high capacity to regenerate NADPH during growth on medium with starch and lactate were found to be the likely cause of this effect. The results lead to the hypothesis that fumonisin production by *A. niger *is regulated by acetyl-CoA.

## Background

*Aspergillus niger *is a versatile filamentous fungus found in the environment all over the world in soil and on decaying plant material and it has been reported to grow on a large number of foods and feeds [1]. At the same time it is a popular production host for industrial fermentations and it is used for production of both organic acids and for indigenous and heterologous enzymes and proteins [2-4]. However, *A. niger *produces various secondary metabolites, and among those also the important mycotoxins fumonisin B_2 _(FB_2_) and ochratoxin A (OTA) [5,6]. Due to the ubiquity of *A. niger*, its production of secondary metabolites is important both from a biotechnological and a food-safety viewpoint.

Secondary metabolites are small molecules that are not directly involved in metabolism and growth. Both plants and fungi are known for producing a large number of chemically diverse secondary metabolites. While the role of some of these metabolites makes sense biologically as inferring an advantage to the producer, e.g. antibiotics, virulence factors, siderophores and pigments, the benefit of others is less obvious or unknown. The general belief is that the secondary metabolites must contribute to the survival of the producer in its environment where it competes with other organisms [7]. Whereas the ability to produce individual secondary metabolites is species-specific, the actual production of secondary metabolites has, in broad terms, been reported to be affected by the developmental stage of the fungus (i.e. conidiation) and intrinsic and extrinsic factors of the environment as substrate (composition, pH, water activity), temperature, light and oxygen availability [8-12].

Fumonisins are a group of secondary metabolites with a highly reduced polyketide-derived structure consisting of a hydrocarbon backbone with an amino group in one end, some methyl groups and two ester-bound side groups consisting of tricarballylic acid moieties. The fumonisin B-series group contains up to three hydroxyl groups and the degree of hydroxylation gives rise to the designations B_1_-B_4_[13,14]. These are classified as mycotoxins as they have been shown to be cytotoxic and carcinogenic [14,15] and fumonisins have been suspected to be involved in oesophageal cancer in South Africa and China [16-19]. Fumonisin production in *Fusarium *spp. has been known since the 1980's [20], while the ability of *A. niger *to produce FB_2 _was just discovered in 2007 based on indications from the genome sequencing projects of *A. niger *ATCC 1015 and CBS 513.88 [6,21,22]. The fumonisin biosynthesis pathway and the gene cluster are partly characterized in *F. verticillioides *and include a polyketide synthase (Fum1), fatty acyl-CoA synthetases (Fum10, Fum16), an aminotransferase (Fum8), a short chain dehydrogenase/reductase (Fum13), cytochome P450 monooxygenases (Fum6, Fum12 and/or Fum15) and a dioxygenase (Fum9) [23]. The expected fumonisin biosynthesis gene cluster in the *A. niger *CBS 513.88 genome contains 14 open reading frames of which a number has similarity to the fumonisin biosynthesis cluster genes in *F. verticillioides *[22]. Although the knowledge of the biosynthesis pathway is incomplete, the expected precursors and cofactors required for production of fumonisins are acetyl-CoA, malonyl-CoA, methionine, alanine, 2-ketoglutarate, O_2 _and NADPH [13].

Due to the late discovery of FB_2 _production in *A. niger*, its ability to produce this metabolite has only been the subject of a few studies. *A. niger *was shown to be a relatively consistent producer of FB_2 _on media such as Czapek yeast autolysate agar (CYA) with 5% NaCl [6,24], yet it was noted that the media that support FB_2 _production in *A. niger *were different from those who were supportive in *F. verticillioides *[6].

To evaluate the potential risk of mycotoxin production in foods and feeds, we explored the influence of substrate on FB_2 _production by *A. niger*. During our screening of food-related carbon sources as glucose, sucrose, lactate, starch and fat we found that lactate, when added to a medium containing starch, could synergistically increase the FB_2 _production compared to either starch or lactate alone. To reveal a biological explanation for this interesting observation, we combined growth physiology studies including measurement of several secondary metabolites with a proteome study.

Proteome studies give information about the capability for metabolic flow in the cell, for maintenance of the cell and for anabolic and catabolic processes. The proteome constitutes the cellular machinery, is energetically expensive to maintain and has a crucial influence on the fitness of the fungus. Protein synthesis and degradation are thus carefully regulated at multiple levels. The use of proteome analysis within studies of filamentous fungi has attracted increasing interest in these years and has recently been reviewed by Carberry and Doyle [25], Kim et al. [26,27] and Andersen and Nielsen [28]. The emergence of fungal genome sequences combined with continuously improved mass spectrometry technologies will further show proteomics as useful for studies in fungal biology.

We report on a 2D gel based proteome study conducted to relate differences in protein levels with differences in secondary metabolites especially FB_2 _production, and with the aim of elaborating on the reasons for an increased FB_2 _production on medium containing starch in combination with lactate.

## Results and discussion

### Growth and secondary metabolite production

**Figure 1:**
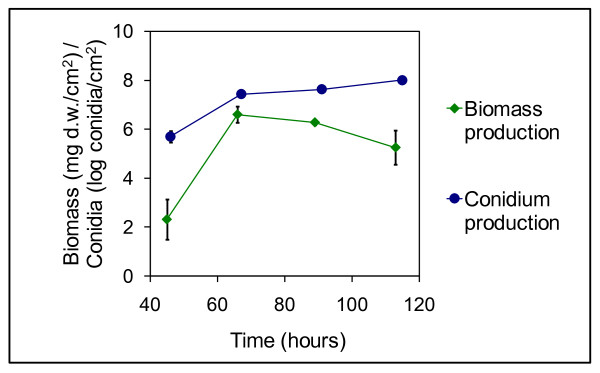
**Growth and conidium production**. Growth measured as biomass production (mg dry weigth/cm^2^) and conidium production (log conidia/cm^2^) by *A. niger *IBT 28144 on medium containing 3% starch. Average values ± standard deviations (n = 3-6).

For these experiments we used a wildtype *A. niger *isolate (*A. niger *IBT 28144) that is able to carry out normal metabolism and synthesis essential for growth and survival in a natural habitat. Additionally it was able to produce both of the two mycotoxins FB_2 _and OTA. With the aim to explore factors that influence secondary metabolism, especially FB_2 _biosynthesis, we used this isolate, grown on the surface of a solid medium and with a moderately rich substrate containing amino acids, nitrate, vitamins, minerals, trace metals and the polysaccharide starch (Czapek Yeast Autolysate agar with saccharose replaced by starch and/or other carbon sources). *A. niger *IBT 28144 grew vigorously under these conditions (Figure 1). Mycelium was observed 20 hours after inoculation and biomass accumulated within 70 hours. Aerial hyphae, the first sign of onset of conidiation, were observed already after 24 hours.

To measure the production of secondary metabolites we used a modified version of a micro-scale extraction procedure [29] that is suitable for detection of a wide array of metabolites. Using plug sampling, the amount of secondary metabolites was determined per surface area of the culture including both metabolites within the cells and metabolites diffusing into the medium. Using this method we detected the following metabolites produced by *A. niger *on starch-containing medium; fumonisin B_2_, fumonisin B_4_, ochratoxin A, ochratoxin alpha, malformin A, malformin C, orlandin, desmethylkotanin, kotanin, aurasperone B, pyranonigrin A and tensidol B.

**Figure 2:**
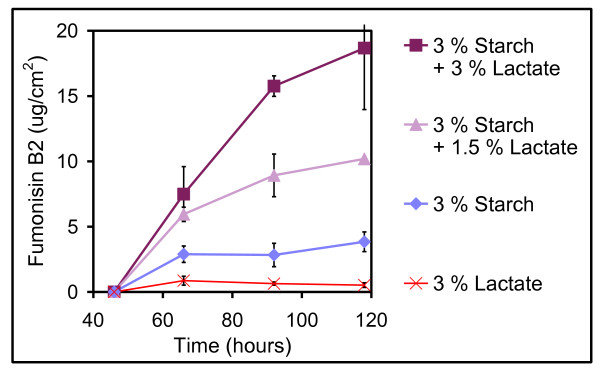
**Fumonisin B_2 _production**. Levels of fumonisin B_2 _(μg/cm^2^) produced by *A. niger *IBT 28144 on media containing 3% lactate, 3 % starch, 3 % starch + 1.5 % lactate and 3 % starch + 3 %  lactate. Average values ± standard deviations (n = 3-18).

**Figure 3:**
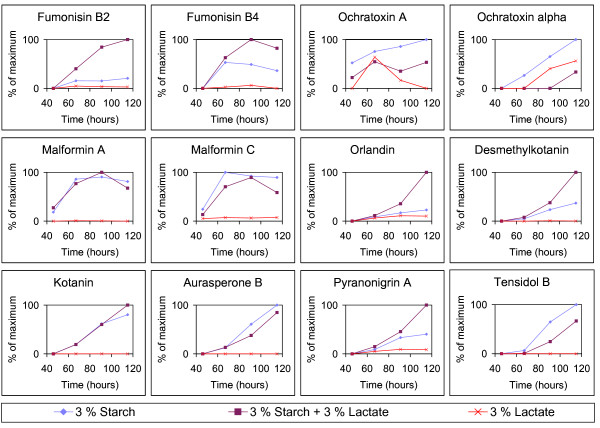
**Secondary metabolite production**. Production of selected secondary metabolites produced by *A. niger *IBT 28144 on media containing 3% starch, 3% starch + 3% lactate and 3% lactate. Data based on average peak area per cm^2 ^(n = 3) calculated as percentage of maximum value obtained for each metabolite.

Presence of lactate, which may be encountered in environments with fermenting microorganisms and especially in fermented food products, was found to increase FB_2 _production considerably when supplied in tandem with starch. The FB_2 _levels detected on media with 3% starch plus 3% lactate were 2-3 times higher than the levels on 3% starch. The differences were significant (95% confidence) at the samplings 66, 92 and 118 hours after inoculation (Figure 2). The stimulating effect of lactate on FB_2 _production seemed to be proportional to the concentration of lactate as 3% starch plus 1.5% lactate resulted in levels intermediate of those containing 3% starch and either no lactate or 3% lactate. Fumonisin B_4_, orlandin, desmethylkotanin and pyranonigrin A were regulated like FB_2 _but only during the later growth phase (Figure 3). Especially the level of the polyketide orlandin was increased synergistically by the combination of starch and lactate. Orlandin, desmethylkotanin and kotanin have very similar polyketide structures and are expected to be part of the same biosynthesis pathway [30], but kotanin was not influenced in the same way as orlandin and desmethylkotanin by presence of starch and lactate. The differential influence of starch and lactate on production of the 12 measured metabolites indicates that secondary metabolism of *A. niger *is not restricted to a common regulation under these conditions. Presence of starch was important for both the growth and the production of secondary metabolites; all were lower on 3% lactate compared to 3% starch with the exception of the ochratoxins that were produced at similar amounts on lactate and starch.

We considered whether the effect of lactate in combination with starch could be due to a specific induction of secondary metabolite synthesis by lactate and if this could constitute some kind of antimicrobial defence. However we found that pyruvate, a product of L-lactate degradation (eq. 1 and 2), had a similar effect (Table 1), which makes an effect of lactate itself unlikely and to a higher degree pointing to an effect of lactate degradation.

**Table 1:** Fumonisin B_2 _production on different carbon sources

Supplemented carbon source	Fumonisin B_2_^1,2 ^(μg/cm^2^)	n^3^
3% Starch	2.89 ± 0.63 ^a^	18
3% Starch + 3% maltose	2.61 ± 0.74 ^a^	3
3% Starch + 3% xylose	2.06 ± 0.28 ^a^	3
3% Starch + 3% lactate	7.49 ± 2.10 ^b^	14
3% Starch + 3% pyruvate	5.06 ± 0.60 ^b^	3
3% Lactate	0.86 ± 0.34 ^c^	15

1) FB_2 _produced (average ± standard deviation) by *A. niger *IBT 28144 after 66-67 hours on media supplemented with the indicated carbon sources.

2) Different letters indicate statistically significant differences using Fisher's least significant difference procedure (95% confidence).

3) Number of replicates.

While it is well known that starch is degraded by extracellular enzymes to maltose and glucose, transported into the cell and then entering glycolysis, we may assume that lactate is transported into the cell by a lactate transporter and mainly metabolized further to pyruvate by a L-lactate dehydrogenase (EC 1.1.1.27) or a L-lactate dehydrogenase (cytochrome) (EC 1.1.2.3), both are predicted to be present in the genome. While the medium with 3% starch + 3% lactate contains approximately the double amount of added carbon source (the yeast extract contains carbon sources as well) compared to the media with 3% starch or 3% lactate alone, it is possible that this is partly counteracted by carbon catabolite repression of the lactate transporter, as the activity of the lactate transporter in yeast, Jen1p, is inversely related to the concentration of repressing sugar [31]. The available energy contributed from 3% lactate is expected to be a bit lower than from 3% starch, as less ATP is generated from 2 lactate (eq. 1 and 2) than from 1 glucose (eq. 3). But, this is based on the assumption that a full conversion of starch to glucose occurs and that glucose is not turned into energy storage metabolites as trehalose or polyols, as it does during liquid culture conditions [32].(1)

In practice, we observed a low biomass production (mg dry weight/cm^2^) on the medium with 3% lactate, while the produced biomass on media containing 3% starch with or without additional 3% lactate was not significantly different. Although the presence of starch was important for both growth and FB_2 _production of *A. niger*, addition of either 3% maltose or 3% xylose to medium containing 3% starch did not further increase the FB_2 _production. The effect of added lactate can consequently not be a simple result of a double amount of carbon source.

### Exploring the proteome

Proteome analysis was conducted in order to identify proteins for which expression levels were altered during growth of *A. niger *on media containing 3% starch (S), 3% starch + 3% lactate (SL) and 3% lactate (L), and if possible relate the identified proteins to the influence on FB_2 _production. The samples for protein extraction were taken 60 hours after inoculation as the FB_2 _production rate was estimated to be highest at this time. In order to document FB_2 _synthesis, FB_2 _production was measured after 58 hours and 66 hours. The FB_2 _synthesis rate was calculated to be (average ± 95% confidence limits, n = 6) 280 ± 140 ng/cm^2^/h on S, 520 ± 90 ng/cm^2^/h on SL and 10 ± 60 ng/cm^2^/h on L. Biomass (dry weight) was measured after 62 hours and was (average ± standard deviations, n = 3) 6.2 ± 0.4 mg/cm^2 ^on S, 6.5 ± 1.0 mg/cm^2 ^on SL and 1.3 ± 0.3 mg/cm^2 ^on L.

**Figure 4:**
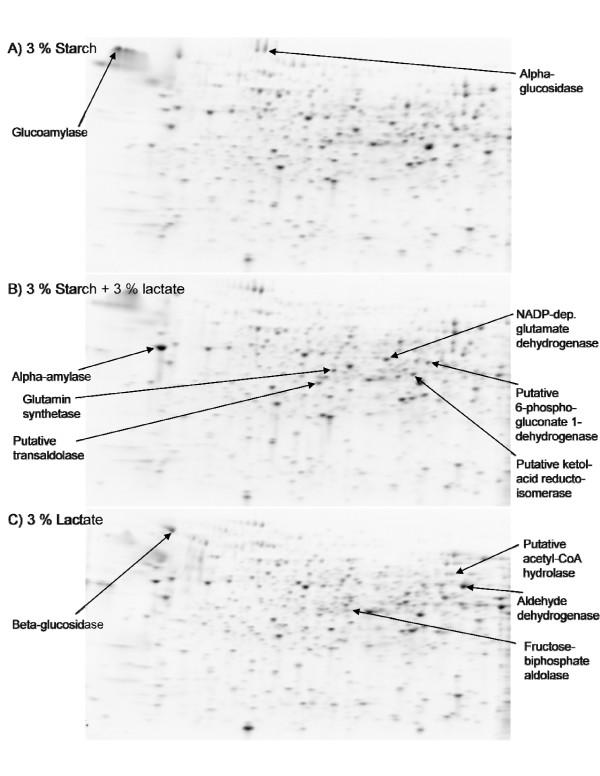
**Example of representative 2D PAGE gels**. 2D PAGE gels of proteins from *A. niger *IBT 28144 after 60 hours growth on media containing 3% starch (top), 3% starch + 3% lactate (middle) and 3% lactate (bottom).

Extracted proteins were separated by two-dimensional polyacrylamide gel electrophoresis (Figure 4). On 18 gels, representing 2 biological replicates and 3 technical replicates of *A. niger *cultures on each of the media S, SL and L, we detected 536-721 spots. With regard to the size of gels and amount of loaded protein, this was comparable to detected spots in other proteome studies of intracellular proteins in *Aspergillus *[33,34]. One protein was present at very high levels on the media containing starch, which was identified as glucoamylase [Swiss-Prot: P69328]. Jorgensen et al. [35] did similarly find this protein to have the highest transcript level of all genes in a transcriptome analysis of *A. niger *on maltose. Because of the volume and diffusion of this spot, the area containing this spot was excluded from the data analysis. About 80% of the spots were matched to spots on a reference gel containing a mixture of all samples. Thus, the total dataset for further analysis consisted of 649 matched spots (see Additional file 1).

**Figure 5:**
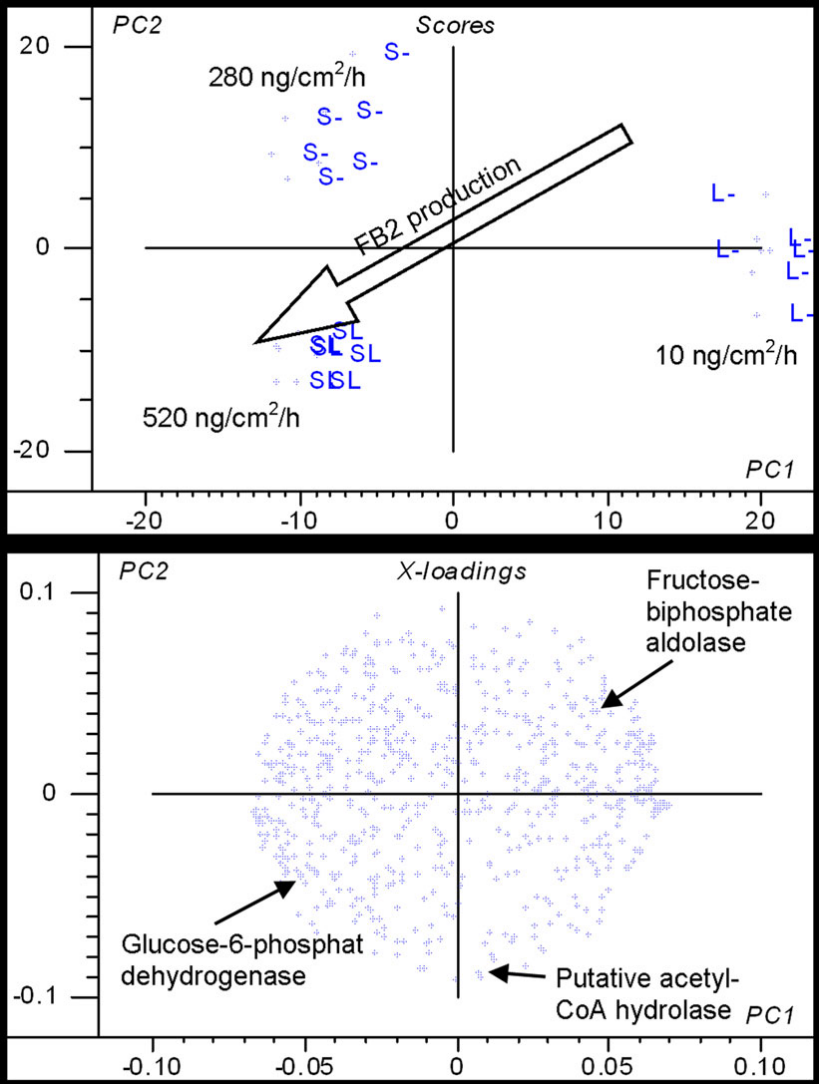
**Illustration of variance in expressed proteins**. Scoreplot (top) and loadingplot (bottom) from a principal component analysis of relative spot volume of all matched spots from the proteome analysis of *A. niger*. Shown is the 1^st ^and 2^nd ^principal component that explain 29% of the variance using validation with systematic exclusion of biological replicates.

Large differences in the proteome of *A. niger *when grown on S, SL and L were evident. A principal component analysis (PCA) clearly separated the gels with proteins from each media into three separate groups (Figure 5). The largest variance in relative spot volume was between samples from media with or without presence of starch (1^st ^component), while the next-largest variance in relative spot volume separated samples from S and SL (2^nd ^component). Statistically, 36% of the spots were present at significantly different levels between two or all three of the treatments (two-sided Students t-test, 95% confidence). Clustering of the 649 spots according to their relative spot volume by consensus clustering [36] resulted in prediction of 39 clusters. More than half of the spots were in clusters with a clear influence of medium on the protein level (18 clusters corresponding to 53% of the spots, Table 2) and 130 spots were in clusters with protein levels affected specifically on SL (cluster (cl.) 4, 7, 8, 35, 36, 37, 38).

**Table 2:** Clusters and interpretation

Description of clusters	Cluster profiles^1^			No. of spots	
				Total	Identified
Higher levels on SL				26	11
Tendency for higher levels on SL				36	16
Lower levels on SL				42	4
Tendency for lower levels on SL				26	16
Higher levels if starch is present				45	3
Lower levels if starch is present				52	0
Higher levels if lactate is present				21	4
Lower levels if lactate is present				35	0
Possibly an effect, instability	Clusters 11, 16, 26, 30			58	3
No effect, instability and noise	Clusters 1, 5, 6, 9, 10, 12, 13, 14, 17, 18, 19, 20, 21, 22, 23, 24, 25, 28, 29, 31, 34			308	1
Total				649	58^2^

1) The graphs show the protein level profiles for selected clusters shown as transformed values between -1 and 1, where 0 indicates the average protein level. The bars give the standard deviations within the clusters.

2) One spot, identified as glucoamylase [Swiss-Prot: P69328], was excluded from the data analysis (see text). Thus the total number of identified spots was 59.

The spots to be identified were selected within clusters with a profile with either distinct or tendency for higher (Table 3) or lower (Table 4) protein levels on SL compared to on S and L as these correlated positively or negatively with FB_2 _production. Also some spots with levels influenced by presence of starch (Table 5) or lactate (Table 6) with either distinct or highly abundant presence on the gels were selected. Spots present at significant different levels between the two or three treatments were preferred. A total of 59 spots were identified using in-gel trypsin digestion to peptides, MALDI TOF/TOF and Mascot searches of retrieved MS/MS spectra to sequences from the databases Swiss-Prot [37] or NCBInr [38]. We did not use any taxonomic restrictions, however all except one protein were confidently identified as *A. niger *(predicted) proteins. One protein (6715) that did not match an *A. niger *protein, probably because it was missed or truncated during sequencing, had a significant match to a protein from *N. crassa *[UniProt: NCU04657]. Only 6 proteins (8 spots) were identified as proteins in the Swiss-Prot database and thus regarded as fully characterised. Otherwise, the proteins were registered in the NCBInr database as it contains the protein entries predicted from the sequencing of the *A. niger *CBS 513.88 genome [22]. Per primo March 2009 the predicted proteome based on this sequencing project contained 13906 predicted proteins of which 47.1% had automatically assigned GO annotations and only 154 proteins had been assigned as manually reviewed in the UniProtKB database [39]. To circumvent the limited number of annotated proteins, we assigned annotations based on sequence similarity to characterised Swiss-Prot proteins in other species using BlastP [40]. A protein annotation was assigned to a protein if it had more than 80% sequence identity to a characterised Swiss-Prot protein and a "putative" annotation to proteins that had 50-80% sequence identity to a characterised protein. Other proteins were assigned a "predicted" function if InterPro domains were predicted using InterProScan [41]. In this way, the identified proteins consisted of 6 (8 spots) fully characterised, 12 with annotation based on sequence similarity, 19 with putative annotation, 13 with predicted function and 6 (7 spots) uncharacterised proteins. The proteins with known functions were mainly involved in processes as: polysaccharide degradation; carbon-, nitrogen- and amino acid metabolism; energy production; protein synthesis, folding and degradation; redox balance and protection against oxidative stress. None of the characterised proteins were known to participate in secondary metabolite biosynthesis. A fatty acid synthase subunit alpha [UniProt: A2Q7B6] was identified, which was present at higher levels on SL compared to on S and L (cl. 35). This protein may contribute to fatty acid biosynthesis to be incorporated in the cell membrane; however it may also be an unrecognised polyketide synthase. One gene coding for a predicted aldo/keto reductase [UniProt: A2Q981] was located adjacent to the predicted FB_2 _biosynthesis cluster in the *A. niger *genome. But this protein was present at higher levels on starch-containing media (cl. 3) and therefore did not correlate with FB_2 _production. Furthermore, proteins involved in secondary metabolite synthesis or processes associated with transport or self-protection are not necessarily located within the clusters. One example is a reductase found to participate in aflatoxin biosynthesis in *A. parasiticus*, although it is not located within the aflatoxin cluster and was regulated differently than the aflatoxin cluster genes [42].

**Table 3:** Identified proteins with higher levels on medium with starch + lactate

Protein	Spot		Identification^1^							Expression	
**Annotation^2^**	**Id**.	**Mass kDa^3^**	**Database**	**Acc. no.**	**Mass kDa**	**pI**	**MP**	**Score**	**SC %**	**Cl. no**.	**Profile**

Alpha-amylase, extracellular	6601	53	NCBInr	A2QL05	55^6^	4.5	5	315	13	35	
Fatty acid synthase subunit alpha	6465	76^4^	NCBInr	A2Q7B6	205	5.9	10	387	5	35	
Glucose-6-phosphate 1-dehydrogenase	6561	59	Swiss-Prot	P48826	59	6.2	3	130	7	35	
Glutamine synthetase	6714	42	NCBInr	A2Q9R3	42	5.5	4	290	16	4	
Heat shock protein Hsp70	6481	73	NCBInr	A2QPM8	70	5.1	5	198	12	4	
Isocitrate dehydrogenase [NADP], mitochondrial, precursor	6644	48	Swiss-Prot	P79089	56	8.5	8	339	14	19	
NADP-dependent glutamate dehydrogenase	6647	48	NCBInr	A2QHT6	50	5.8	6	382	18	4	
Predicted 2-nitropropane dioxygenase	6737	41	NCBInr	A2QKX9	38^6^	5.7	4	112	17	35	
Predicted glucose-methanol-choline (Gmc) oxidoreductase	6515	65	NCBInr	A2R501	65	5.4	6	373	18	35	
Predicted methyltransferase	6810	36	NCBInr	A2QNF3	37	5.9	5	200	21	30	
Predicted NADH cytochrome b5 reductase	6693	44	NCBInr	A2R2Z2	46	5.4	6	530	20	4	
Predicted ubiquitin conjugating enzyme	7044	17	NCBInr	A2QDZ9	17	5.5	2	105	18	4	
Putative 6-phosphogluconate dehydrogenase, decarboxylating	6660	47	NCBInr	Q874Q3	55	5.9	9	527	27	35	
Putative aconitate hydratase, mitochondrial	6472	75	NCBInr	A2QSF4	84	6.2	7	278	11	35	
Putative heat shock protein Ssc1, mitochondrial	6487	71	NCBInr	A2R7X5	72	5.6	5	282	9	4	
Putative histidine biosynthesis trifunctional protein	6413	101^5^	NCBInr	A2QAS4	92	5.4	2	147	3	4	
Putative inositol-1-phosphate synthase	6573	57	NCBInr	A2QV05	58	5.7	2	62	4	35	
Putative ketol-acid reductoisomerase, mitochondrial	6730	41	NCBInr	A2QU08	45^6^	8.9	8	467	17	35	
Putative oxoglutarate dehydrogenase	6408	101^5^	NCBInr	A2QIU5	119	6.3	10	349	8	35	
Putative peroxiredoxin pmp20, peroxisomal membrane	7000	22	NCBInr	A2R0G9	19	5.4	8	610	54	4	
Putative peroxiredoxin Prx1, mitochondrial	6944	28	NCBInr	A2QIF8	23	5.2	5	224	22	4	
Putative pyruvate dehydrogenase E1 component subunit alpha, mitochondrial precurser	7028	18^4^	NCBInr	A2QPI1	45	7.6	2	160	7	30	
Putative transaldolase	6787	38	NCBInr	A2QMZ4	36	5.6	5	319	20	4	
Putative transketolase	6471	75	NCBInr	Q874Q5	75	6.0	6	246	11	4	
Thioredoxin reductase	6680	45	NCBInr	A2Q9P0	39	5.2	6	449	22	4	
Uncharacterised protein	6965	26	NCBInr	A2QDU1	19	5.4	3	147	15	4	
Uncharacterised protein	6591	55	NCBInr	A2QDX8	57	5.8	10	601	23	4	
Uncharacterised protein	6592	55	NCBInr	A2QDX8	57	5.8	10	717	25	4	
Uncharacterised protein	7059	16	NCBInr	A5ABN7	26	10.3	2	145	14	35	
Uncharacterised protein	7092	13^5^	NCBInr	A2QSA8	13	5.2	2	249	35	4	

List of identified proteins showing from left to right: Protein name, spot id and observed mass on gels, database, UniProt KB accession number, expected mass and isoelectric point (pI), number of matching peptide sequences (MP), Mowse Score (Score) and sequence coverage (SC), cluster and graph showing protein levels (average relative spot volume ± standard deviation) on media containing 3% starch (left/blue), 3% starch + 3% lactate (middle/purple) and 3% lactate (right/red).

1) Identification was based on Mascot MS/MS Ion Search using sequence data from the databases Swiss-Prot or NCBInr. Protein matches with significant (p < 0.05) Mowse Scores and ≥ 2 matching peptides were regarded as possible candidates for identification.

2) Annotation of uncharacterised proteins was based on sequence homology to characterised Swiss-Prot proteins using BlastP. Proteins were given a full annotation if they had > 80% sequence identity to a characterised Swiss-Prot protein or a putative annotation if they had 50-80% sequence identity to a characterised protein. Remaining proteins were assigned a "predicted" function if InterPro domains were predicted using InterProScan.

3) Observed mass on reference gel calibrated with molecular weight standards (14.4-97.4 kDa).

4) The spot is most likely a fragment as the retrieved peptides were localized in one of the ends of the protein sequence.

5) Mass above or below calibration range

6) The protein is predicted to contain a signal peptide.

7) The protein is predicted to be glycosylated.

**Table 4:** Identified proteins with lower levels on medium with starch + lactate

Protein	Spot		Identification^1^							Expression	
**Annotation^2^**	**Id**.	**Mass kDa^3^**	**Database**	**Acc. no.**	**Mass kDa**	**pI**	**MP**	**Score**	**SC %**	**Cl. no**.	**Profile**

Aldehyde dehydrogenase	6605	53	Swis-Prot	P41751	54	6.0	10	908	34	37	
Aldehyde dehydrogenase	6615	52	Swis-Prot	P41751	54	6.0	7	646	20	38	
Beta-glucosidase 1 precurser	6360	130^5^	NCBInr	Q30BH9	94	4.7	5	267	6	36	
Fructose-biphosphate aldolase	6766	39	NCBInr	A2QDL0	40	5.5	8	697	28	37	
Predicted estherase/lipase/thioesterase	6451	82	NCBInr	A2QTP5	84	5.4	9	543	18	37	
Predicted fumaryl-acetoacetate hydrolase	6663	47	NCBInr	A2QIN6	45	5.2	6	611	24	38	
Predicted glutathione-S-transferase	6952	27	NCBInr	A2R874	24	5.1	5	391	31	37	
Predicted NAD-dependant epimerase/dehydratase	6707	43	NCBInr	A2R992	38	5.7	7	397	26	38	
Predicted ribose/galactose isomerase	7035	18	NCBInr	A2QCB3	17	7.7	7	593	61	36	
Predicted Zn-containing alcohol dehydrogenase	6718	42	NCBInr	A2QAN5	39	5.8	4	298	19	38	
Putative 1-aminocyclopropane-1-carboxylate deaminase	6715	42	NCBInr Cross sp.	Q7S3B7	39	5.8	2	115	11	38	
Putative glutamate carboxypeptidase-like	6609	53	NCBInr	A2QY36	53	5.2	12	811	29	38	
Putative HIT family protein 1	7091	13^5^	NCBInr	A2QLN7	15	6.3	3	227	40	37	
Putative H-transporting two sec tor ATPase subunit F, vacuolar	7083	14	NCBInr	A2QCE6	14	5.3	4	340	44	37	
Putative NADH ubiquinone reductase, 40 kDa subunit, mitochondrial	6738	41	NCBInr	A2QSH0	43	6.7	5	307	17	38	
Putative peroxiredoxin pmp20, peroxisomal membrane	7031	18	NCBInr	A2R6R3	18	5.6	5	431	37	38	
Superoxide dismutase Cu-Zn, cytoplasmic	7046	17	Swiss-Prot	A2QMY6	16	5.9	5	323	38	36	
Ubiquitin-like protein	7113	11^5^	NCBInr	A2QKN1	9	5.8	5	272	60	37	
Uncharacterised protein	7002	21	NCBInr	A2QLX7	20	6.1	7	592	55	8	
Uncharacterised protein	7074	15^4^	NCBInr	A2QBG0	34	5.1	6	609	24	38	

See legend and notes to table 3.

**Table 5:** Identified proteins with levels influenced by presence of starch

Protein	Spot		Identification^1^							Expression	
**Annotation^2^**	**Id**.	**Mass kDa^3^**	**Database**	**Acc. no.**	**Mass kDa**	**pI**	**MP**	**Score**	**SC %**	**Cl. no**.	**Profile**

Alpha-glucosidase, extracellular	6354	151^5^	Swiss-Prot	P56526	109	5.1	7	497	10	2	
Glucoamylase isoform G1, glycosylated	6000	130^5^	Swiss-Prot	P69328	69^6,7^	4.3	5	308	10	-	-
Predicted aldo/keto reductase	6781	38	NCBInr	A2Q981	37	6.0	5	335	17	3	
Pyruvate decarboxylase	6540	61	NCBInr	A5AA75	63	6.3	6	412	15	3	
Translation elongation factor 2	6836	35^4^	NCBInr	A2QD36	94	6.5	6	556	7	11	

See legend and notes to table 3.

**Table 6:** Identified proteins with levels influenced by presence of lactate

Protein	Spot		Identification^1^							Expression	
**Annotation^2^**	**Id**.	**Mass kDa^3^**	**Database**	**Acc. no.**	**Mass kDa**	**pI**	**MP**	**Score**	**SC %**	**Cl. no**.	**Profile**

Alpha-glucfosidase, extracellular	6355	157^5^	Swiss-Prot	P56526	109	5.1	3	147	4	27	
Predicted NMR-like protein	6783	38	NCBInr	A2R745	34^6^	5.2	3	225	14	27	
Putative acetyl-CoA hydrolase, glycosylated	6533	62	NCBInr	A2R8G9	58^7^	6.0	5	253	10	27	
Putative NADH ubiquinone reductase, 31 kD subunit	6888	32	NCBInr	A2QWS1	32	7.7	2	104	8	27	

See legend and notes to table 3.

A throughout tendency was that many of the proteins influenced by the combination of starch and lactate in the medium were likely to affect either the acetyl-CoA level or the NADPH level as discussed below.

### Regulation of central metabolic enzymes

**Figure 6:**
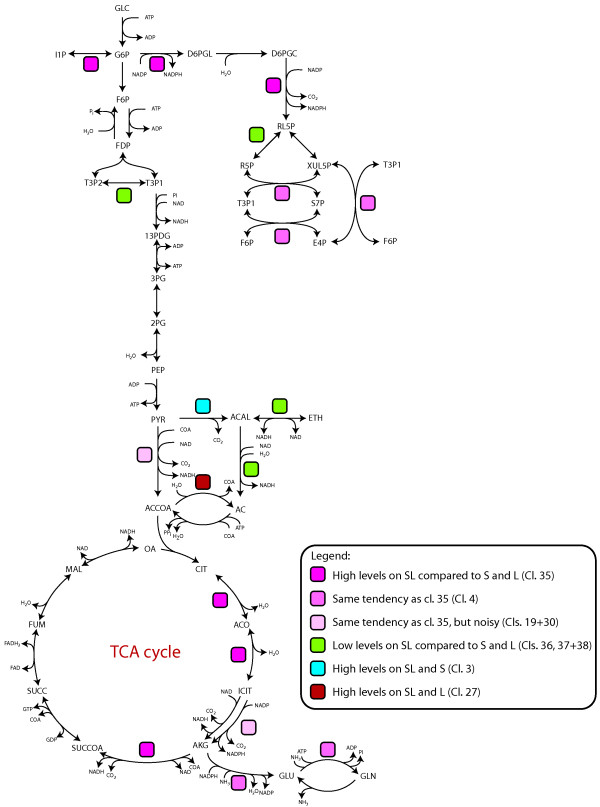
**Identified proteins within the primary metabolism**. Pathway map showing an outline of the glycolysis, the pentose phosphate pathway, pyruvate metabolism, the tricarboxylic acid cycle and ammonium assimilation enzymes with the identified proteins indicated. Modified from map of *A. niger *metabolism published by Andersen et al [68]. 13PDG: 1,3-bisphospho-D-glycerate, 2PG: 2-phospho-D-glycerate, 3PG: 3-phospho-D-glycerate, AC: acetate, ACAL: acetaldehyde, ACCOA: acetyl coenzyme A, ACO: cis-aconitate, AKG: 2-oxoglutarate, CIT: citrate, D6PGC: 6-phospho-D-gluconate, D6PGL: d-glucono-1,5-lactone 6-phosphate, E4P: D-erythrose 4-phosphate, ETH: ethanol, F6P: beta-D-fructose 6-phosphate, FDP: beta-D-fructose 1,6-bisphosphate, FUM: fumarate, G6P: alpha-D-glucose 6-phosphate, GLC: alpha-D-glucose, GLN:L-glutamine, GLU: L-glutamate, I1P:1D-inositol 3-phosphate, ICIT: isocitrate, MAL: (S)-malate, OA: oxaloacetate, PEP: phosphoenolpyruvate, PYR: pyruvate, R5P: D-ribose 5-phosphate, RL5P: D-ribulose 5-phosphate, S7P: sedoheptulose 7-phosphate, SUCC: succinate, SUCCoA: succinyl coenzyme A, T3P1: D-glyceraldehyde 3-phosphate, T3P2: glycerone phosphate (DHAP), XUL5P:D-xylulose 5-phosphate.

The identified proteins appeared to include several important enzymes in the primary metabolism (Figure 6). Glucose 6-phosphate 1-dehydrogenase [Swiss-Prot: P48826] and a putative 6-phosphogluconate dehydrogenase [UniProt: Q874Q3], the first (rate-controlling) and third enzyme in the oxidative part of the pentose phosphate pathway (PPP) were present at higher levels on SL (cl. 35). They both reduce NADP to NADPH, and these enzymes are believed to be the main source of NADPH regeneration in the cell [43-46]. Additionally three enzymes in the non-oxidative part of the PPP were identified. A putative transketolase [UniProt: Q874Q5] and a putative transaldolase [UniProt: A2QMZ4] had tendencies for higher levels on SL (cl. 4). A predicted ribose/galactose isomerase [UniProt: A2QCB3], presumably with ribose 5-phosphate isomerase activity, was present at lower levels on SL (cl. 36). Lower level of this enzyme, responsible for synthesis of ribose 5-phosphate required for the biosynthesis of some amino acids, nucleotides, and coenzymes, indicates that the PPP was optimised to NADPH regeneration rather than to nucleotide synthesis on SL. One glycolysis enzyme, fructose-biphosphate aldolase [UniProt: A2QDL0], had tendency for lower level on SL (cl. 37), which is in good agreement with a higher activity of the PPP. Those enzymes identified downstream of pyruvate, the entry point of lactate into metabolism, were either clearly present at higher levels on SL or had the tendency for higher level. This included a putative pyruvate dehydrogenase (E1 subunit alpha) [UniProt: A2QPI1] (cl. 30) and the three enzymes in the tricarboxylic acid (TCA) cycle converting citrate to isocitrate, the irreversible step from isocitrate to 2-oxoglutarate, and from 2-oxoglutarate to succinyl-CoA. The first and the third TCA cycle enzyme, a putative aconitate hydratase [UniProt: A2QSF4] and a putative 2-oxoglutarate dehydrogenase [UniProt: A2QIU5], was clearly present at higher levels on SL (cl. 35), while NADP-dependant isocitrate dehydrogenase [Swiss-Prot: P79089] had a tendency for higher level but with a noisy profile (cl. 19). One enzyme that occurred at higher level when lactate was present in the media (cl. 27) was a putative acetyl-CoA hydrolase [UniProt: A2R8G9]. This enzyme has been designated to catalyse the hydrolysis of acetyl-CoA to acetate, but may rather posses CoA transferase activity between succinyl-, propionyl- and acetyl-CoA and the corresponding acids [47]. In yeast, acetyl-CoA hydrolase is involved in trafficking of acetyl-CoA across membranes in the form of acetate and thus is expected to be important for regulation of the acetyl-CoA level [48,49].

To summarize, higher levels of the enzymes in the PPP that generate NADPH during growth on SL compared to on S and L indicate an increased ability to regenerate NADPH when the NADP:NADPH ratio is increased. The higher levels of the enzymes in the metabolism of pyruvate after pyruvate enters mitochondria on SL and the higher levels of putative acetyl-CoA hydrolase in presence of lactate indicate an increased amount of carbon passing through acetyl-CoA during growth on SL.

### Regulation of enzymes influencing the NADPH level

A remarkable requirement for NADPH on SL medium is pointed out by the simultaneous effect on several of the relatively few enzymes that contribute to NADPH regeneration. We found glucose 6-phosphate dehydrogenase, putative 6-phosphogluconate dehydrogenase, NADP-dependent isocitrate dehydrogenase and putative ketol-acid reductoisomerase [UniProt: A2QUO8], an enzyme in isoleucine, leucine and valine biosynthesis, to be present at higher levels on SL. Regulation of these enzymes is probably due to an increased NADP:NADPH ratio. The activity of the first enzyme, glucose 6-phosphate dehydrogenase, is known to be regulated by NADP:NADPH levels [50]. Larochelle et al. [51] showed in yeast that transcription of the corresponding gene was also affected by the NADPH level and they attributed this to a transcription factor Stb5. The yeast cell regulates the metabolism to counteract a high NADP:NADPH ratio by up-regulating the PPP and down-regulating glycolysis [51], which neatly corresponds to the changes we have observed in these pathways.

*A. niger *needs a supply of NADPH for several anabolic and biosynthetic processes as well as for protection against oxidative stress. A supply of NADPH is for example required in order to utilize nitrate as nitrogen source, since the enzyme that converts nitrate to nitrite, nitrate reductase, uses NADPH as cofactor [44].

On SL, we observed higher levels of enzymes involved in fatty acid biosynthesis, ammonium assimilation and protection against oxidative stress, those activities may increase the NADP:NADPH ratio [52]. As mentioned previously, we observed a higher level of a fatty acid synthase subunit alpha on SL (cl. 35) that requires NADPH in order to catalyse the biosynthesis of fatty acids. We also identified NADP-dependant glutamate dehydrogenase [UniProt: A2QHT6] involved in ammonium assimilation and thioredoxin reductase [UniProt: A2Q9P0] that utilises NADPH to reduce thioredoxin during conditions with oxidative stress; both had tendencies for higher levels on SL (cl. 4). Furthermore, the polyketide synthase involved in FB_2 _biosynthesis uses NADPH as cofactor [13] and that may also affect the NADP:NADPH ratio.

These results show a clear tendency towards increased NADPH turnover and regeneration during growth on SL.

### Relation between regulated proteins and FB_2_ biosynthesis

The identified proteins regulated on SL were mainly enzymes in the primary metabolism and other processes that likely affect the intracellular levels of acetyl-CoA or NADPH. The higher FB_2 _production on SL is thus most likely a result of changes in the metabolism due to lactate degradation. Acetyl-CoA is a precursor for production of FB_2 _as well as for other polyketide-derived metabolites [13]. High level of acetyl-CoA during growth on SL may thus be what drives the high FB_2 _production. This is supported by the observation that pyruvate had a similar effect as lactate on FB_2 _production. A good ability to regenerate NADPH when the NADP:NADPH ratio is increased may be an important prerequisite for the high FB_2 _production on SL.

However, the effect of added lactate to a medium containing starch on FB_2 _production was dramatic and not expected to be solely precursor-driven. Further, the 12 secondary metabolites measured in this study, which include polyketides, non-ribosomal peptides and polyketide-derived alkaloids, were affected differently by the presence of starch and lactate and a pattern reflecting the biosynthetic origin of the metabolites was not evident. This supports that the influence of lactate in combination with starch on FB_2 _production is regulatory rather than an effect solely driven by abundance of precursors. We hypothesise that the FB_2 _production, when induced, could be regulated globally according to the nutrient/energy state. As a central compound in metabolism, carefully regulated and compartmentalised, acetyl-CoA may be a candidate for this [53]. Acetyl-CoA has been shown to be able to affect transcription in vitro [54]. In yeast, it has been suggested that transcription of the inositol 1-phosphate synthase gene, *ino1*, is influenced by the acetyl-CoA level during conditions with high levels of energy-rich metabolites [55]. In accordance, we identified a putative inositol-1-phosphate synthase [UniProt: A2QV05] among the proteins with higher levels on SL medium (cl. 35). Inositol-1-phosphate synthase is the first and rate-controlling enzyme in the inositol biosynthesis pathway and converts glucose 6-phosphate into inositol 1-phosphate. Inositol is incorporated into phosphatidylinositol that in turn is a precursor of sphingolipids and inositol polyphosphates, required for a diverse set of processes that include glycolipid anchoring of proteins, signal transduction (regulation of chromatin remodeling and transcription), mRNAexport and vesicle trafficking [56,57]. Acetyl-CoA is also a substrate for protein acetylation by protein acetylases, and acetylation can influence both gene expression and protein activity [58]. In *A. parasiticus *there has been observed a correlation between initiation and spread of histone acetylation in the aflatoxin gene promoters and the initiation of aflatoxin gene expression [59]. Another study of *A. nidulans *has shown that genetic deletion of a histone deacetylase caused elevated gene expression and enhanced production of sterigmatocystin and penicillin [60]. The same study demonstrated that treatment with histone deacetylase inhibitors could enhance production of some secondary metabolites by *Penicillium expansum *and *Alternaria alternata*, indicating that histone acetylation and deacetylation have a role in regulation of secondary metabolite production in a broad range of fungal genera.

Secondary metabolite synthesis can be subject to multiple regulatory mechanisms. Regulation of fumonisin B_1 _biosynthesis in *F. verticillioides *has been found to be complex with several positive and negative regulators and influenced by nitrogen, carbon and pH [12,61]. Corresponding to our results, fumonisin B_1 _production in *F. verticillioides *has been shown to be induced by the presence of starch [62]. However, *F. verticillioides *and *A. niger *are widely different physiologically and genetically, thus production and regulation of fumonisin biosynthesis are not expected to be identical [6].

During conditions where *A. niger *spends resources on producing extracellular enzymes for degradation of plant tissue and starch, protection against other microorganisms competing for nutrients would be beneficial. Fumonisin B_1 _has been shown to have antifungal activity against species as *Alternaria alternata*, *Penicillium expansum*, *Botrytis cinerea *and *Fusarium graminearum *[63], thus FB_2 _could be expected to have a similar effect. Increased production of FB_2 _during conditions with high acetyl-CoA level may thus have evolved because antifungal activity was advantageous to *A. niger *as a way to protect the nutrient sources in the environment.

## Conclusions

Our results show that lactate, when supplemented in a rich substrate containing nitrate and starch, can increase the FB_2 _production in *A. niger*. Based on the identified proteins within the central metabolism, we suggest this to be due to changes in the balance of intracellular metabolites towards a higher level of carbon passing through acetyl-CoA and a high capacity to regenerate NADPH. Given that the FB_2 _biosynthesis genes are induced, the results indicate that the availability of precursors and NADPH has a large influence on production of FB_2_. The production of certain other secondary metabolites was affected in a similar fashion as FB_2 _by lactate (fumonisin B_4_, orlandin, desmethylkotanin and pyranonigrin A), while other secondary metabolites were not (ochratoxin A, ochratoxin alpha, malformin A, malformin C, kotanin, aurasperone B, tensidol B). Consequently, as these metabolites were affected differently by the presence of starch and lactate, they must be regulated differently in *A. niger*.

We find it likely that the influence of starch and lactate/pyruvate on FB_2 _production is part of a global regulation inferred by the nutrient/energy state and propose that this could be through the action of acetyl-CoA. Whether, if and how, acetyl-CoA affects gene transcription or activity of enzymes in the FB_2 _biosynthesis pathway could be the scope of relevant, future studies.

It remains to be seen whether production of secondary metabolites in other species of filamentous fungi is increased by presence of starch and lactate. The effect of starch and lactate in combination may be relevant to be aware of for starch-containing foods and feeds where fungi occur concurrently with lactic acid fermentation, which could be the case in low-fat mould-fermented sausages, in fermented vegetable products and in silage. Technologically, the obtained knowledge of substrate influence on production of specific secondary metabolites could be beneficial, as lactate or other carbon sources could be used to increase metabolite production during industrial fermentation.

## Methods

### Strain

*A. niger *IBT 28144 (CBS 101705) was obtained from the IBT culture collection and maintained on silica gel. The culture was used after two successive inoculations on Czapek Yeast Autolysate agar (CYA), incubated 7 days in dark at 25°C.

### Media

Media were modified from CYA and contained per L: 5 g Yeast extract (Biokar Diagnostics, Beauvais, France); 3 g NaNO_3_; 1 g K_2_HPO_4_; 0,5 g KCl; 0,5 g MgSO_4_·7H_2_O; 0,01 g FeSO_4_·7H_2_O; 0,01 g ZnSO_4_·7H_2_O; 0,005 g CuSO_4_·5H_2_O and 20 g agar (Sobigel, VWR - Bie & Berntsen A/S, Herlev, Denmark). Soluble potato starch, 60% potassium L-lactate solution, maltose monohydrate, D-xylose and/or sodium pyruvate (all Sigma Aldrich, St. Louis, Missouri, USA) were added according to the indicated percentages in w/v. Lactate, maltose, xylose and pyruvate and the remaining ingredients were sterilised separately, at 121°C for 15 min., cooled to 60°C before the ingredients were mixed, adjusted to pH 5.5 with sterile filtered 2 M KOH or 5 M HCl and poured into petri dishes.

### Inoculation and incubation

Conidium suspensions were prepared in spore suspension media (0.50 g Tween 80, 0.50 g agar to 1 L water), filtrated through Miracloth (Merck KGaA, Darmstadt, Germany) to remove mycelium fragments and adjusted to 10^6 ^conidia/ml. Each agar plate was surface inoculated with 10^5 ^conidia using a drigalsky spatula. Incubation was in dark at 25°C.

### Determination of growth

Biomass production was determined in triplicate for surface inoculated cultures on agar plates covered with a 0.45 μm polycarbonate membrane (Isopore™, Millipore, Billerica, Massachusetts, USA). The whole mycelium was collected and the dry weight was determined after drying at 100°C for 20-24 h.

### Determination of conidium production

Eight agar plugs (4 mm in diameter) were dispensed in 4 ml peptone water (1 g peptone (Difco, BD, Franklin Lakes, New Jersey, USA) to 1 l destilled water) and replicate measures of the conidium concentration were determined in a Thoma counting chamber for triplicate cultures.

### Extraction of secondary metabolites

The method described by Smedsgaard [29] with some modifications was used for secondary metabolite extraction. A sample of 8 agar plugs (4 mm in diameter) taken randomly from the plate was extracted with 1 ml methanol/dichloromethane/ethyl acetate (v/v/v 1:2:3) containing 1% (v/v) formic acid for 60 min using ultrasonication. The extract was transferred to a new vial and the solvent evaporated. The agar plug sample was re-extracted with 0.8 ml 75% methanol in water for 60 min using ultrasonication and the extract combined with the dry extract of first extraction. The residues were re-dissolved by whirley mixing followed by 10 min ultrasonication and the extracts were filtrated through 0.45 μm PTFE filters.

### LC-MS and HPLC-FLD for determination of secondary metabolites

LC-MS was performed on an Agilent 1100 LC system (Agilent Technologies, Santa Clara, California, USA) with a 40°C, 50 mm × 2 mm i. d., 3 μm, Luna C18 II column (Phenomenex, Torrance, California, USA). The LC system was coupled to a single quadropole mass detector (LC/MSD VL, Agilent technologies) with an atmospheric pressure ionisation source and to a 200-700 nm diode array detector. A sample volume of 3 μl was injected and eluted at a flow rate of 0.3 ml/min using a water-acetonitrile gradient system starting from 15% acetonitrile that was increased linearly to 100% in 20 min and with a holding time of 2 min. Water and acetonitrile were buffered with 20 mM formic acid and 5 mM ammonium formiate (only water). The ion source was operated in positive mode with a capillary voltage at 3000 V and detection was done in full scan from m/z 100-1000, a peak width of 0.1 min and a cycle time of 1.06 sec. HPLC-FLD was performed on a similar LC system coupled to a fluorescence detector. Water and acetonitrile were buffered with 50 mM trifluoroacetic acid (TFA). Excitation and emission wavelengths were 333 nm and 460 nm respectively. Chemstation (Agilent) was used for data collection and evaluation. Detection was based on the extracted ion chromatogram of the ions [M+H]^+ ^or [M+NH_3_]^+ ^or fluorescence emission chromatograms (Table 7). Standards were used for confirmation of identity if available. Otherwise the identity was confirmed by presence of characteristic ions or adducts in the MS spectrum and characteristic UV absorbance spectrum. Quantification of FB_2 _was based on a calibration curve created from dilutions of a fumonisin B_2 _standard (50.1 μg/ml, Biopure, Tulln, Austria) at levels from 0.5 to 25 μg/ml. The remaining metabolites were semi-quantified based on peak areas, calculated in percentage of highest average peak area value of triplicates within the study.

**Table 7:** Detection parameters for selected *A. niger *secondary metabolites

Metabolite		Detection			Confirmation		
		Method^1^		Rt^2^	**Std**.	MS ions and adducts^1^	UV peak absorption wavelengths^3^
Fumonisin B_2_	[6]	MS	[M+H]^+ ^= m/z 706	9.6	×	[M+Na]^+ ^= m/z 728	End^4^
Fumonisin B_4_	[24]	MS	[M+H]^+ ^= m/z 690	10.5	-	-	End^4^
Ochratoxin A	[5]	FLD	Excitation: 333 nm, emission: 460 nm	10.3	×	-	216 nm (100), 250 nm (sh), 332 nm (20) [69]
Ochratoxin alpha	[70]	FLD	Excitation: 333 nm, emission: 460 nm	7.1	×	-	216 nm (100), 235 nm (sh), 248 nm (sh), 336 nm (22) [69]
Malformin A_1_	[71]	MS	[M+NH3]^+ ^= m/z 547	10.5	×	[M+H]^+ ^= m/z 530, [M+Na]^+ ^= m/z 552	End^4^
Malformin C	[72]	MS	[M+NH3]^+ ^= m/z 547	10.9	×	[M+H]^+ ^= m/z 530, [M+Na]^+ ^= m/z 552	End^4^
Orlandin	[73]	MS	[M+H]^+ ^= m/z 411	7.5	-	[M+Na]^+ ^= m/z 433	Similar to kotanin
Desmethyl-kotanin	[30]	MS	[M+H]^+ ^= m/z 425	9.3	-	[M+Na]^+ ^= m/z 447	Similar to kotanin
Kotanin	[30]	MS	[M+H]^+ ^= m/z 439	11.4	×	[M+Na]^+ ^= m/z 461	208 nm (100), 235 nm (sh), 296 nm (sh), 308 nm (47), 316 nm (sh) [69]
Aurasperone B	[74]	MS	[M+H]^+ ^= m/z 607	11.5	-	[M+Na]^+ ^= m/z 629	233 nm (68), 270 nm (sh), 280 nm (100), 318 nm (24), 331 nm (24), 404 nm (15)[75]
Pyranonigrin A	[76]	MS	[M+H]^+ ^= m/z 224	1.7	-	[M+NH4]^+ ^= m/z 241, [M+Na]^+ ^= m/z 246	210 nm (100), 250 nm (51), 314 nm (68) [77]
Tensidol B	[78]	MS	[M+H]^+ ^= m/z 344	9.1	-	[M+Na]^+ ^= m/z 366	206 nm (100), 242 nm (44) [78]

List of secondary metabolites included in this study with reference of their production in *A. niger*. Detection method and retention time, available standards used for confirmation (marked by x) and additional MS and UV spectral information used for confirmation.

1) Values obtained from Antibase 2007 (Wiley, Hoboken, New jersey, USA).

2) Retention time in respective LC systems (OTA and OT-alpha analysis on separate HPLC system).

3) Parenthesis values are absorption in percent of maximum absorption, sh denotes a shoulder.

4) End: End absorption (< 200 nm).

### Sampling for proteome analysis

Duplicate samples for proteome analysis were taken from surface inoculated cultures on agar plates covered with a 0.45 μm polycarbonate membrane (Isopore™, Millipore). The whole mycelium mass was collected and frozen in liquid nitrogen.

### Protein extraction

The method described by Kniemeyer et al. [64] with few modifications was used for protein extraction. The mycelium was homogenised with mortar and pestle under liquid nitrogen and 100 mg of the homogenate was collected. The protein was precipitated with acetone added with 13.3% (w/v) trichloroacetic acid and 0.093% (v/v) 2-mercaptoethanol at -20°C for 24 hours followed by centrifugation at 20.000 × g in 15 min at 4°C. Pellet was washed twice in acetone with 0.07% (v/v) 2-mercaptoethanol and air-dried for 10 min. Pellet was suspended in 600 μl sample buffer containing 7 M urea, 2 M thiourea, 2% (w/v) CHAPS, 0.8% (v/v) ampholytes (Bio-Lyte 3/10, Bio-Rad, Hercules, California, USA), 20 mM DTE and 20 mM Tris (Tris-HCl buffer pH 7.5). The solution was incubated for 1 hour at 20°C and ultrasonicated for 10 min. The sample was centrifuged at 17.000 × g for 30 min, and the supernatant was collected and stored at -80°C. Protein concentration was determined using a 2-D Quant kit (GE Healthcare, Uppsala, Sweden).

### 2D polyacrylamide gel electrophoresis

Isoelectric focusing was done using immobilised pH gradient strips (11 cm, pH 4-7, ReadyStrip™, Bio-Rad). A sample volume corresponding to either 40 μg (image analysis gels) or 100 μg (preparative gels) protein was diluted to a total volume of 200 μl in a rehydration buffer consisting of 7 M urea; 2 M thiourea; 2% (w/v) CHAPS; 0.5% (v/v) ampholytes (Bio-Lyte 3/10, Bio-Rad); 1% (w/v) DTT and 0.002% (w/v) bromophenol blue. Rehydration was done at 250 V for 12 hours at 20°C. Focusing was done at an increasing voltage up to 8000 V within 2 1/2 hour and hold until 35 kVh was reached, with a maximal current of 50 μA/IPG strip. The voltage was hold at 500 V until the IPG strips were frozen at -20°C. The IPG strips were equilibrated in buffer containing 6 M urea, 30% (w/v) glycerol, 2% (w/v) SDS in 0.05 M Tris-HCl buffer pH 8.8. First, the cysteines in the sample were reduced in equilibration buffer added with 1% (w/v) DTT for 15 min, and when alkylated in equilibration buffer added with 4% (w/v) iodoacetamide for 15 min. PAGE was done at 200 V in 10-20% gradient gels (Criterion Tris-HCl Gel, 10-250 kD, 13.3 × 8.7 cm, Bio-Rad) using an electrode buffer containing 25 mM Tris, 1.44% (w/v) glycine and 0.1% (w/v) SDS. Image analysis gels were fixed in 50% (v/v) ethanol, 7% (v/v) acetic acid two times for 30 min and stained over night in SYPRO Ruby Protein Gel Stain (Invitrogen, Life Technologies, Carlsbad, California, USA). The gels were washed in 10% (v/v) ethanol, 7% (v/v) acetic acid for 30 min. and two times in Milli-Q water (Millipore) for 5 min. The gels were visualized with a CCD camera (Camilla fluorescence detection system, Raytest, Straubenhardt, Germany) equipped with excitation and emission filters and with an exposure time of 100 ms. Images were saved as 16 bit tif-files. Preparative gels were fixed in 15% (w/v) ammoniumsulphate, 2% (v/v) phosphoric acid, 18% (v/v) ethanol in water and stained with Coomassie Brilliant blue (0.02% (w/v) Brilliant blue G in fixing buffer) overnight and washed two times in Milli-Q water. Gels were prepared in triplicate for each biological sample for image analysis gels and a reference gel containing an equal mixture of all samples was included. A molecular weight standard (14.4 - 97.4 kDa, BioRad) was applied to the reference gel before PAGE for mass calibration.

### Image analysis

Images were imported, inverted and analyzed with Imagemaster 2D platinum v. 5 (GE Healthcare). Spot detection parameters were adjusted for optimal spot detection (smooth = 2; min. area = 30; saliency = 20) and the spots were quantified as the relative spot volume (percent spot volume) within each gel. The spots from each gel were paired with detected spots on a reference gel containing a mixture of all samples. Matching of gels was done automatically after selection of a landmark spot in each gel.

### Statistical analysis

Statistical differences in relative spot volumes between the treatments were determined by two-sided Students t-tests (H_0_: μ_1 _= μ_2_, H_A_: μ_1 _≠ μ_2_) using Imagemaster 2D platinum. The null hypothesis was rejected if t_df = 2 _≤ 4.303 (95% confidence).

Statistical analysis of FB_2 _production was done using Statgraphics Plus v. 4.0 (StatPoint Inc., Herndon, Virginia, USA).

### Principal component analysis

Principal component analysis was done using Unscrambler v. 8.0 (Camo Process AS, Oslo, Norway). The dataset consisted of 18 gels (samples) and 649 spots (variables) and corresponding relative spot volumes. All variables were centred and weighted by (standard deviation)^-1^. Validation was based on systematic exclusion of samples corresponding to a biological replicate.

### Cluster analysis

Cluster analysis was done using the Matlab clustering algorithm "ClusterLustre" described by Grotkjær et al [36]. The relative spot volumes were transformed to Pearson distances prior to clustering (results in values between -1 and 1, where 0 indicates the average expression level). Cluster solutions with K = 3-50 clusters were scanned with 20 repetitions. For each repetition the most likely number of clusters was determined by the Bayesian Information Criteria.

### In-gel digestion of proteins

In-gel digestion was done according to Shevchenko et al. [65] with some minor modifications: The protein spots were excised from Coomassie stained gels loaded with 100 μg protein. A piece of gel without staining was used as a negative control. The gel pieces were cut into approx. 1 mm^3 ^pieces and washed twice for 15 min., first with water and second with water/acetonitrile 1:1 (v/v). The gel particles were then washed in acetonitrile to dehydrate the gel (they shrunk and became white). A volume of 10 mM dithiotreitol (DTT) in 100 mM NH_4_HCO_3 _to cover the gel pieces was added and the proteins were reduced for 45 min at 56°C. After cooling, the DTT solution was replaced by the same volume of 55 mM iodoacetamide in 100 mM NH_4_HCO_3 _and the reduced proteins were alkylated for 30 min. in the dark. The gel pieces were then washed with water, water/acetonitrile 1:1 (v/v) and acetonitrile to dehydrate the gel. Ice-cold digestion buffer containing 12.5 ng/μl trypsin in 50 mM NH_4_HCO_3 _was added to the gel pieces in a volume just sufficient to rehydrate the gel (5-10 μl). After 45 min incubation on ice bath the unabsorbed digestion buffer was removed and replaced by 20 μl of 50 mM NH_4_HCO_3 _buffer to cover the gel pieces. The proteins were digested overnight at 37°C. The buffer solution with protein digest was recovered and kept at -20°C.

### Micropurification of peptides and loading on MALDI target

The peptide solutions were purified on nano-scale reversed-phase columns prior to mass spectrometric analysis by the method described by Gobom et al [66]. The columns were prepared by loading a few μl slurry of a reversed phase chromatographic medium (Poros R2 10 μm, Applied Biosystems) dissolved in acetonitrile into a partially constricted GelLoader pipette tip. The column was packed by applying pressure with a syringe giving a column height of 4-10 mm and equilibrated with 1% TFA. The peptide digest was loaded onto the column and desalted by washing with 1% TFA. The peptides were eluted with matrix solution containing 5 μg/μl *α*-cyano-4-hydroxycinnamic acid in 70% acetonitrile and 0.1% TFA directly in one droplet onto the MALDI target (Opti-TOF^® ^384 Well MALDI Plate Inserts, Applied Biosystems, California, USA).

### MALDI TOF/TOF tandem MS

MALDI peptide mass spectra and MS/MS spectra of selected peptides were obtained on a 4800 Plus MALDI TOF/TOF™ Analyzer (Applied Biosystems). External mass calibration was done using a tryptic digest of beta-lactoglobolin (m/z 837.48 and 2313.26) and in some cases peaks from trypsin auto-digestion peptides (m/z 842.51 and 2211.12) were used for internal calibration of the peptide mass spectra. MS and MS/MS mass spectra were obtained at a laser intensity of 3000 and 3600 respectively. Peak lists were generated with an in house macro (in the Protein Research Group at Department of Biochemistry and Molecular Biology, University of Southern Denmark) using Data Explorer (Applied Biosystems) and converted to .mgf files containing the combined data from MS and MS/MS spectra for a sample.

### Protein identification

Mascot MS/MS Ions Search (Matrix Science [67]) was used to search for matching protein sequences within the databases Swiss-Prot (Swiss Institute of Bioinformatics [37]) or NCBInr (National Center for Biotechnology Information [38]). The search parameters were: enzyme digestion with trypsin, no taxonomic restriction, carbamidomethyl (C) as fixed modification, oxidation (M) as variable modification, [M+1]^+ ^peptide charge state, monoisotopic mass values, unrestricted protein mass, ± 70 ppm peptide mass tolerance, ± 0.6 Da fragment mass tolerance, maximum 1 missed cleavage pr. peptide. Protein matches to *Aspergillus niger *proteins and with significant (p < 0.05) Mowse Scores were regarded as possible candidates for identification. The candidate(s) were further inspected for number of matching peptides (=2), the mass accuracy of the matching peptides, the sequence coverage and distribution of matching peptides in the obtained sequences. The reported miscleavage sites were inspected for presence of amino acids that affect the action of trypsin (proline, glutamic acid and aspartic acid or additional lysine/arginine). Finally the molecular weight and isoelectric point of the obtained protein match were compared to those observed on the gels. From samples with low intensity, peptides from keratin and trypsin were erased if necessary.

### Protein annotation

Annotation of uncharacterised proteins was based on sequence similarity to characterised Swiss-Prot proteins using BlastP [40]. Proteins were given a full annotation if they had more than 80% sequence identity to a characterised Swiss-Prot protein or a putative annotation to proteins if they had 50-80% sequence identity to a characterised protein. Other proteins were assigned a "predicted" function if InterPro domains were predicted using InterProScan (European Bioinformatics Institute [41]).

## Authors' contributions

LMS participated in design of the study, carried out the experimental work, the statistical and multivariate analysis and prepared the manuscript. RL participated in design of the study, contributed to the proteome analysis and revised the manuscript. MRA carried out the cluster analysis, participated in protein annotation and interpretation and revised the manuscript. PVN and JCF participated in design of the study and revision of the manuscript. All authors read and approved the final manuscript.

## Supplementary Material

Additional file 1 **Protein expression data**. Additional file 1.xlsx (an excel file) contains relative spot volumes for spots detected and matched to a reference gel in the 2D gel based proteome analysis of *A. niger *IBT 28144 on the three media containing 3% starch (S), 3% starch + 3% lactate (SL) and 3% lactate (L). B1-B6 denotes the biological replicate, R1-R2 the electrophoresis run and Gel 1-21 the gel number. Click here for file
